# Systems-level actions of luteolin in female reproductive disorders: from molecular mechanisms to clinical translation

**DOI:** 10.3389/fphar.2026.1768006

**Published:** 2026-02-25

**Authors:** Lenah S. Binmahfouz

**Affiliations:** Department of Pharmacology and Toxicology, Faculty of Pharmacy, King Abdulaziz University, Jeddah, Saudi Arabia

**Keywords:** female reproductive disorders, inflammation, luteolin, oxidative stress, PI3K/AKT/PTEN

## Abstract

Female reproductive disorders represent a major global health challenge. Despite their clinical heterogeneity, these conditions share core pathological mechanisms including oxidative stress, chronic inflammation, hormonal imbalance, metabolic dysfunction, extracellular matrix remodeling, and dysregulated cell survival. Current therapies rarely target these interconnected processes, underscoring the need for multi-pathway modulators. Luteolin, a dietary flavone, has emerged as a promising candidate due to its regulatory effects on redox balance, NF-κB/MAPK signaling, PI3K/AKT/PTEN pathways, TGF-β/Smad-mediated fibrosis, and estrogen and progesterone receptor activity. Preclinical and mechanistic evidence demonstrates luteolin’s benefits across major reproductive disorders. In PCOS, it improves insulin sensitivity, supports ovulatory function, modulates hepatic and ovarian gene expression, and influences gut microbiota. In endometriosis, it disrupts epithelial-macrophage crosstalk, reduces chemokine-driven inflammation, and inhibits angiogenesis and lesion growth. In leiomyomas, luteolin attenuates fibrosis and normalizes apoptotic and TGFB1/PI3K/PTEN signaling. Protective effects on ovarian reserve in primary ovarian insufficiency, anti-inflammatory and anti-ferroptotic actions in endometritis, and suppression of sFlt-1 and HIF-1α in preeclampsia further highlight its relevance to reproductive pathology. Anticancer and chemosensitizing effects have also been reported in ovarian, cervical, and endometrial cancers. Although clinical translation is constrained by poor solubility and bioavailability, emerging nanocarrier and prodrug strategies markedly improve luteolin’s pharmacokinetic profile. Human studies of luteolin-based formulations support anti-inflammatory and antioxidant effects consistent with reproductive disease mechanisms. Overall, luteolin represents a multi-target pharmacological candidate with translational potential in gynecologic and endocrine disorders, warranting further optimization and early-phase clinical investigation.

## 1 Introduction

Female reproductive health is influenced by a complex interplay of endocrine, metabolic, inflammatory, and tissue-remodeling processes. Disruptions in these systems can lead to a range of chronic gynecological conditions that affect millions of women worldwide (Sharami et al., 2025). These disorders include polycystic ovary syndrome (PCOS), endometriosis, uterine leiomyomas, primary ovarian insufficiency (POI), endometritis, and gynecologic cancers. Despite their clinical heterogeneity, these disorders share convergent pathological drivers, including oxidative stress, chronic inflammation, hormonal dysregulation, metabolic dysfunction, aberrant extracellular matrix (ECM) remodeling, and impaired apoptosis (As-Sanie et al., 2025; Fartushok et al., 2025; Kumar and Ramanarayanan, 2025). Current medical and surgical treatments primarily address symptoms but do not effectively target the mechanisms central to disease progression and recurrence (Azam et al., 2025). As a result, there is a significant need for multi-target interventions that can simultaneously restore balance across redox, immune, endocrine, and metabolic systems.

Luteolin, a naturally occurring flavone found in various herbs, vegetables, and botanical preparations, has emerged as a promising candidate for integrated therapeutic modulation (Zhu et al., 2024). Its chemical structure, characterized by a planar 3′,4′,5,7-tetrahydroxyflavone scaffold, confers strong antioxidant, anti-inflammatory, anti-fibrotic, and metabolic-regulatory properties (Hasnat et al., 2024; Ibrahim, 2025). Mechanistically, luteolin engages multiple molecular networks, including the Nrf2/ARE antioxidant axis, NF-κB/MAPK inflammatory cascades, PI3K/AKT/PTEN metabolic-survival pathway, TGF-β/Smad fibrotic signaling, and estrogen (ER) and progesterone (PR) receptor-mediated hormonal regulation (Thiruvengadam et al., 2021; Khan et al., 2024). This broad mechanistic spectrum provides a compelling rationale for luteolin’s consistent benefits across diverse reproductive disorders.

Extensive preclinical evidence supports these mechanistic insights, demonstrating that luteolin restores key cellular and endocrine functions across reproductive models. In PCOS, it enhances insulin sensitivity, normalizes ovulatory function, and reduces ovarian oxidative damage (Dutta et al., 2025; Yuan et al., 2025). For endometriosis, luteolin disrupts macrophage-lesion interactions, decreases angiogenesis, and suppresses chemokine-driven inflammation (Woo et al., 2021). In cases of uterine leiomyomas, it inhibits fibrotic remodeling, restores apoptotic balance, and reduces inflammation in the myometrium (Binmahfouz et al., 2025). Furthermore, luteolin helps preserve ovarian reserve in chemotherapy- or toxin-induced POI (Pan et al., 2025), and protects epithelial integrity in cases of endometritis by reducing cytokine-mediated and ferroptotic injury (Gao et al., 2024). Additionally, luteolin exhibits antiproliferative, anti-metastatic, and chemosensitizing effects in ovarian, cervical, and endometrial cancers (Li et al., 2023; Zhao et al., 2023; Pei et al., 2024). Together, these findings demonstrate that luteolin targets the intersecting molecular pathways that drive multiple reproductive disorders.

Despite a strong mechanistic and preclinical foundation, the clinical application of luteolin has been limited by its poor aqueous solubility, extensive phase II conjugation, and low oral bioavailability (Wang et al., 2024; Lv et al., 2025). However, recent advances in formulation technologies, such as nanocrystals, polymeric micelles, β-cyclodextrin-metal-organic frameworks, and SNEDDS/S-SNEDDS systems, and metabolically activated prodrugs, have significantly improved its dissolution, stability, metabolic protection, and systemic exposure (Batool et al., 2020; Miao et al., 2021; Wu et al., 2024; Yang et al., 2024). Alongside these developments, international patent activity has increased, indicating growing commercial and scientific interest (Wei and Ma, 2014; Song Kwon and Sun-woo, 2021; Jia et al., 2024). Despite this progress, most filings remain in early developmental stages and primarily emphasize nutraceutical combinations rather than engineered pharmaceutical formulations.

Emerging human studies provide encouraging translational support, showing that luteolin, administered primarily as ultramicronized PEA-luteolin, reduces key inflammatory cytokines (IL-6, IL-1β, TNF-α), mast-cell mediators, and oxidative stress biomarkers (Cordaro et al., 2020; De Luca et al., 2022; Di Stadio et al., 2022). These effects align closely with mechanisms driving reproductive inflammation, metabolic dysfunction, and fibrotic remodeling. Ongoing Phase II trials (Buchanan, 2025) further underscore luteolin’s therapeutic potential.

Therefore, this review aims to synthesize current mechanistic, preclinical, and translational evidence on luteolin across major female reproductive disorders and to evaluate its potential as a multi-target therapeutic candidate. It also integrates advances in formulation science, regulatory developments, and patent activity, and outlines a translational roadmap to guide future clinical development.

## 2 Methodology

A comprehensive literature search was conducted to identify published evidence on the chemistry, pharmacokinetics, mechanisms of action, preclinical activity, clinical data, formulations, and regulatory aspects of luteolin in female reproductive disorders. Electronic databases searched included PubMed®, Scopus®, Web of Science™, ScienceDirect®, Google Scholar®, and patent repositories such as Google Patents, USPTO, CNIPA, and KIPO. Search terms combined (“luteolin” OR “flavone”) with reproductive indications (“PCOS,” “endometriosis,” “uterine fibroids/leiomyomas,” “primary ovarian insufficiency,” “endometritis,” “gynecologic cancers”) and mechanistic keywords (“oxidative stress,” “inflammation,” “fibrosis,” “PI3K/AKT/PTEN,” “Nrf2,” “NF-κB,” “TGF-β,” “drug delivery systems”). Eligible studies included peer-reviewed *in vitro*, *in vivo*, translational, clinical studies, and patents published in English from database inception through October 2025. Exclusion criteria were non-scientific sources, conference abstracts without full data, and studies unrelated to reproductive or mechanistic relevance. Patents were grouped by indication and formulation type. Clinical reports were evaluated for dose, duration, formulation matrix, biomarkers, and safety outcomes. This approach ensured a structured and comprehensive synthesis of luteolin’s therapeutic potential in female reproductive medicine.

## 3 Luteolin overview

### 3.1 Chemical structure and classification

Flavonoids represent a major class of plant polyphenols characterized by a C6-C3-C6 skeleton. They are categorized into several classes, including flavones, flavonols, flavanones, isoflavones, flavanols, and anthocyanidins (Stachelska et al., 2025). This classification is based on the oxidation state and substitution pattern of the central heterocyclic C-ring (Hasnat et al., 2024). Luteolin, also known as 3′,4′,5,7-tetrahydroxyflavone, is a representative member of the flavone subclass. Flavones are defined by a C2 = C3 double bond and a carbonyl group at C4 within the C-ring. The presence of four hydroxyl groups at positions 3′and 4′on the B-ring and 5 and 7 on the A-ring underlies luteolin’s potent antioxidant and radical-scavenging properties (Abou Baker, 2022; de Aguiar et al., 2025). These functions are achieved through hydrogen donation and resonance stabilization of phenoxyl radicals (Ibrahim, 2025). The conjugated C2 = C3 bond and 4-oxo group facilitate electron delocalization within the molecule, enhancing luteolin’s affinity for enzymes and receptors through hydrogen bonding and π–π stacking interactions (Abdrabou et al., 2024). Beyond its redox properties, structural and computational studies indicate that luteolin’s planar flavone scaffold allows direct interaction with ATP-binding pockets of key kinases such as PI3K and ERK, and with nuclear receptors including ERα and PPARγ (Lee et al., 2015). These molecular interactions enable luteolin to modulate both metabolic and hormonal signaling, highlighting its importance in oxidative and endocrine regulation.

### 3.2 Natural dietary and herbal sources

Luteolin is widely distributed in edible plants, culinary herbs, and beverages, contributing substantially to dietary flavonoid intake. Prominent natural sources include celery (*Apium graveolens*), parsley (Petroselinum crispum), thyme (Thymus vulgaris), peppermint (Mentha piperita), oregano (Origanum vulgare), green pepper (Capsicum annuum), and rosemary (Rosmarinus officinalis) (Manzoor et al., 2017; Shen et al., 2022). According to the U.S. Department of Agriculture (USDA) Database for the Flavonoid Content of Selected Foods, Release 3.3 (2018), luteolin content typically ranges between 1 and 6 mg/100 g in most herbs and vegetables. Complementary population-based data from the National Health and Nutrition Examination Survey (NHANES) indicate that the median daily intake of luteolin among U.S. adults is approximately 0.3 mg/day (interquartile range: 0.1–0.8 mg/day), with higher consumption noted in Mediterranean and East Asian dietary patterns (Yao and Zhou, 2024). Recent food-science evidence shows that cooking and preparation methods strongly influence flavonoid stability and release. Thermal processes such as boiling or prolonged heating can result in up to 50% reduction in total phenolics, including luteolin, whereas controlled enzymatic or fermentative processing can enhance the aglycone content, thereby improving intestinal absorption and bioavailability (González-Coria et al., 2024). These findings emphasize that food preparation and processing methods play a critical role in determining the effective bioactive exposure to luteolin from dietary sources.

### 3.3 Pharmacokinetics and bioavailability

A clear understanding of luteolin’s pharmacokinetic behavior is essential for translating its preclinical promise into therapeutic applications in reproductive disorders. Overall, luteolin displays suboptimal pharmacokinetic performance characterized by limited aqueous solubility, low oral exposure, and rapid systemic clearance (Wang et al., 2024). Its chemical scaffold, particularly the 3′,4′-dihydroxy arrangement and conjugated double bond, enhances antioxidant capacity yet inherently restricts solubility and absorption (Ren et al., 2024). Current evidence further indicates modest systemic availability of the active aglycone and limited tissue penetration, including in reproductive organs. These pharmacokinetic constraints contribute to difficulty achieving *in-vivo* concentrations comparable to those seen in mechanistic *in-vitro* studies. As a result, considerable research has shifted toward improving luteolin delivery through nanocarriers, phospholipid complexes, and prodrug approaches designed to enhance solubility, stability, and bioavailability (Ibrahim, 2025).

**FIGURE 1:**
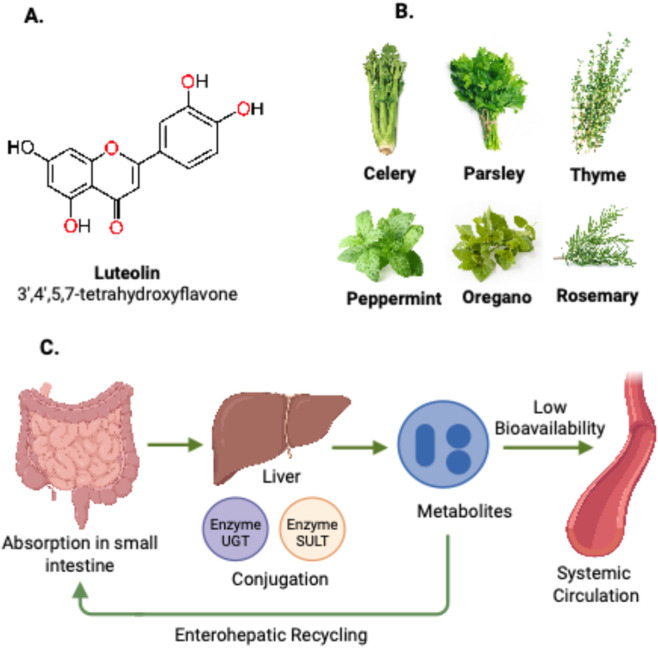
Chemical structure, natural sources, and metabolic fate of luteolin. **(A)** Chemical structure of luteolin, a 3′,4′,5,7-tetrahydroxyflavone characterized by a core flavone backbone bearing four hydroxyl groups. **(B)** Natural dietary and herbal sources rich in luteolin, including celery, parsley, thyme, peppermint, oregano, and rosemary. **(C)** Metabolism and circulatory pathway of luteolin. After absorption in the small intestine, luteolin undergoes extensive phase II conjugation in the liver, mediated by UDP-glucuronosyltransferases (UGTs) and sulfotransferases (SULTs), generating circulating metabolites with low systemic bioavailability. Conjugated metabolites are excreted into bile and undergo enterohepatic recycling, allowing partial reabsorption of luteolin.

After oral intake, luteolin is absorbed in the small intestine and undergoes extensive phase II conjugation during first-pass metabolism. These reactions are primarily mediated by UDP-glucuronosyltransferases (UGTs) and sulfotransferases (SULTs), which generate major circulating metabolites such as luteolin-7-O-glucuronide and luteolin-3′-O-glucuronide (Lv et al., 2025). Consequently, plasma contains predominantly glucuronidated and sulfated conjugates, while the aglycone appears only in trace amounts. These conjugates enter the bile and participate in enterohepatic recycling, which, together with gut microbiota–mediated deconjugation, shapes systemic exposure and contributes to inter-individual variability (Xiong et al., 2023). Double-peak plasma profiles observed in rodents support this recycling phenomenon (Sarawek et al., 2008). Absolute oral bioavailability is generally modest, around 4%–26% depending on formulation and dosing regimen (Wang et al., 2024). This highlights the major influence of solubility and first-pass metabolism on systemic availability. As a result, *in-vivo* concentrations may fall below thresholds required to reproduce potent mechanistic effects demonstrated *in vitro*. Figure 1 summarizes luteolin’s structure, dietary origins, and metabolic pathway.

Luteolin’s physicochemical profile is dominated by its extremely low aqueous solubility, with only 0.02–0.03 mg/mL dissolving in water at 27 °C (Rajhard et al., 2021). Despite adequate membrane permeability, this low solubility places luteolin within a Biopharmaceutics Classification System (BCS) class II–type compound, where dissolution (not permeability) is the primary barrier to oral uptake (Chen et al., 2022). Limited dissolution in gastrointestinal fluids restricts the fraction of luteolin that becomes available for absorption, contributing to its low and highly variable oral exposure. These physicochemical constraints provide the rationale for developing formulation strategies specifically aimed at enhancing solubility and dissolution to improve systemic availability.

## 4 Luteolin in female reproductive disorders

Luteolin has been investigated across a wide spectrum of female reproductive conditions, ranging from endocrine-metabolic dysfunctions to inflammatory, fibrotic, infectious, and malignant disorders. Despite their diverse etiologies, these diseases converge on several recurring pathological themes, including oxidative stress, chronic inflammation, hormonal imbalance, aberrant extracellular matrix deposition, and dysregulated cell survival. Because luteolin influences multiple biological processes simultaneously, it is well positioned to target these shared mechanisms. This section provides a disorder-focused overview of luteolin’s biological effects, summarizing the experimental evidence across reproductive models.

### 4.1 Polycystic ovary syndrome

Polycystic ovary syndrome is a common endocrine and metabolic disorder affecting 8%–13% of women of reproductive age. It is characterized by chronic anovulation, hyperandrogenism, insulin resistance, and ovarian morphological changes (Almhmoud et al., 2024). The pathogenesis of PCOS is linked to oxidative stress, inflammation, and alterations in steroid hormone production (Rosenfield and Ehrmann, 2016). Given its multifactorial nature, therapeutics that simultaneously target oxidative stress, inflammation, steroidogenic imbalance, and insulin resistance are of growing interest. Luteolin’s broad antioxidant, anti-inflammatory, and metabolic regulatory actions make it a strong candidate for modulating several key pathways implicated in PCOS pathophysiology.

In the widely used letrozole + high-fat diet rat model, luteolin administration significantly improved reproductive and metabolic features (Huang and Zhang, 2021). Luteolin restored estrous cyclicity, improved follicular maturation, and normalized hormone profiles (↓LH, ↓testosterone, ↑FSH, ↑estradiol). Metabolically, it reduced hyperinsulinemia and improved insulin sensitivity (↓HOMA-IR), accompanied by enhanced ovarian antioxidant defenses. These findings suggest that luteolin supports ovarian function in PCOS by improving both endocrine balance and metabolic responsiveness, likely through modulation of insulin signaling and oxidative status within the ovary.

Building on these findings, Dai et al. (2025) extended the therapeutic relevance of luteolin to PCOS complicated by obesity, a phenotype that amplifies metabolic stress and worsens reproductive outcomes. In an obese rat model of PCOS, luteolin markedly improved estrous cyclicity, restored ovarian morphology, enhanced glucose tolerance, and reduced dyslipidemia (↓TG, ↓TC, ↓LDL-c). Beyond reproductive improvements, luteolin showed strong metabolic benefits by attenuating hepatic steatosis and reversing the expression of key liver genes involved in mitochondrial function, insulin signaling, and lipid metabolism (including UQCRC2, IRS2, NFIX, and ALDH6A1). Metagenomic profiling further demonstrated that luteolin increased gut microbial diversity and shifted the microbiota toward a healthier composition, notably increasing Bacteroidota and decreasing Firmicutes. These findings highlight luteolin’s multi-system role in PCOS, demonstrating that its benefits extend beyond ovarian function to include hepatic metabolic regulation and restoration of gut microbiota homeostasis—processes highly relevant in obesity-exacerbated PCOS.

Beyond endocrine-metabolic PCOS models, luteolin also mitigates environmentally induced PCOS-like ovarian dysfunction. Bisphenol A (BPA), a well-established endocrine disruptor, induces ovarian injury through oxidative stress, mitochondrial dysfunction, and dysregulation of PCOS-associated genes (Urbanetz et al., 2023). In Chinese Hamster Ovary (CHO) cells, luteolin reduced reactive oxygen species (ROS) accumulation, prevented mitochondrial depolarization, and suppressed apoptosis (Sudhakaran et al., 2024). In zebrafish models, luteolin normalized follicular maturation, increased superoxide dismutase (SOD) activity, improved gonadosomatic index, and decreased follicular atresia. It also downregulated inflammatory and PCOS-associated genes (TNF-α, IL-1β, TOX3, DENND1A) and improved acetylcholinesterase activity (Sudhakaran et al., 2024).

### 4.2 Primary ovarian insufficiency

Primary ovarian insufficiency (POI), historically referred to as premature ovarian failure (POF), affects 1%–3% of women under 40 and arises from accelerated follicular loss or dysfunction (Touraine et al., 2024). Causes include oxidative stress, apoptosis, cytotoxic chemotherapeutic agents, autoimmune injury, and DNA damage (Wu J. et al., 2025). In cyclophosphamide-induced POI, luteolin improved serum levels of estradiol, progesterone, AMH, LH, and FSH, restoring endocrine function (Pan et al., 2025). Histologically, luteolin preserved follicular architecture, reduced granulosa cell apoptosis, and increased antioxidant enzyme activity. Markers of oxidative injury and DNA damage (MDA, 4-HNE, 8-OHdG) were significantly reduced. High-level mechanistic evidence suggests luteolin supports DNA repair processes, reduces oxidative injury, and maintains granulosa cell viability, factors essential for ovarian reserve preservation.

### 4.3 Endometriosis

Endometriosis is a chronic, estrogen-dependent inflammatory disorder characterized by the ectopic implantation and growth of endometrial tissue. It affects 10%–15% of reproductive-aged women and up to half of infertile women (Tsamantioti and Mahdy, 2025). The disorder is sustained by a complex interplay of hormonal responsiveness, persistent inflammation, aberrant immune-lesion communication, angiogenesis, and progressive fibrotic remodeling (As-Sanie et al., 2025). These processes collectively promote the survival, invasion, and vascularization of ectopic lesions. Luteolin has demonstrated multifaceted therapeutic actions across both *in vitro* and *in vivo* models of endometriosis. In human 12Z endometriotic epithelial cells, luteolin suppresses proliferation and induces apoptosis through activation of caspase-dependent pathways (Woo et al., 2021). Beyond its direct cytotoxic effects on ectopic epithelial cells, luteolin disrupts key immune-lesion interactions that support disease progression. It significantly reduces the expression of chemokines such as CCL2 and CCL5, thereby limiting the recruitment of pro-endometriotic macrophages. In macrophage co-culture systems, luteolin inhibits M2-like polarization, a macrophage phenotype known to promote angiogenesis, fibrosis, and lesion maintenance. Consistent with these immune-modulatory actions, luteolin downregulates VEGF and matrix metalloproteinases (MMP-2 and MMP-9), attenuating neovascularization and extracellular matrix remodeling (Woo et al., 2021).

Complementary evidence from Park et al. (2019) demonstrates that luteolin targets fundamental proliferative and survival pathways within endometriotic cells. Using human endometrial/ectopic cell lines (VK2/E6E7 and End1/E6E7) and a mouse auto-implantation model, the study showed that luteolin markedly reduces lesion growth *in vivo*. Mechanistically, luteolin induces G0/G1 cell-cycle arrest through downregulation of critical cell-cycle regulators, including CCNE1, CDK2, and CDK4. It simultaneously inhibits PI3K/Akt and MAPK signaling—core pathways that support cell survival, proliferation, and resistance to apoptosis in endometriotic tissue. This coordinated suppression of proliferative and pro-survival networks leads to increased apoptosis and significant reduction in implant size *in vivo*.

### 4.4 Endometritis

Endometritis is a bacterial inflammation of the endometrial lining affecting 2%–5% of women following childbirth or uterine instrumentation. It contributes to infertility, pelvic pain, and impaired implantation (Yan et al., 2025). Disease progression involves microbial invasion, excessive cytokine production, disruption of epithelial barrier proteins, oxidative stress, and in severe cases, ferroptotic injury to endometrial tissue (Tabeeva et al., 2024). Emerging evidence demonstrates that luteolin exerts broad protective actions across multiple experimental models of infectious endometritis. In a *Staphylococcus aureus*–induced mouse model, luteolin markedly attenuated uterine inflammation, reducing neutrophil infiltration, edema, and tissue injury (Gao et al., 2024). Pro-inflammatory cytokines (TNF-α, IL-1β, IL-6) were significantly suppressed, while epithelial barrier integrity was restored through upregulation of tight-junction proteins ZO-1 and occludin. Luteolin also inhibited ferroptosis by lowering malondialdehyde (MDA) and ferrous iron (Fe^2+^), while increasing glutathione and GPX4 levels, highlighting its ability to mitigate oxidative epithelial damage.

Complementary findings from an LPS-induced endometritis model further support luteolin’s anti-inflammatory and antioxidant potential (Shaukat et al., 2024). Therapeutic administration of luteolin substantially reduced uterine histopathological injury and suppressed pro-inflammatory cytokines (IL-1β, IL-6, TNF-α), while increasing the anti-inflammatory cytokine IL-10. Luteolin enhanced antioxidant defenses (↑SOD1, ↑CAT, ↑GPx1; ↓MDA, ↓ROS) and modulated innate immune signaling by upregulating TLR4 expression yet simultaneously inhibiting NF-κB activation. Activation of Nrf2 further contributed to oxidative stress resolution. Collectively, these data indicate that luteolin counteracts LPS-triggered endometrial injury through coordinated modulation of TLR4-associated Nrf2 and NF-κB pathways. Luteolin also demonstrates therapeutic activity against biofilm-associated endometritis, which is typically more resistant to antimicrobial therapy (Zhang et al., 2022). In *T. pyogenes*, a major opportunistic pathogen causing suppurative uterine infections, luteolin dispersed mature biofilms and significantly inhibited the expression of biofilm-related genes (luxS, plo, rbsB, lsrB). In a rat endometritis model induced by glacial acetic acid followed by *Trueperella pyogenes* inoculation, luteolin treatment reduced uterine inflammation and symptom severity, highlighting its potential role in managing refractory biofilm-associated infections.

### 4.5 Uterine leiomyomas

Uterine leiomyomas (fibroids) are the most common benign gynecologic tumors, affecting 20%–40% of reproductive-aged women and up to 70% by age 50 (Bulun et al., 2025). Their growth is driven by estrogen and progesterone signaling, chronic oxidative stress, inflammation, and excessive ECM accumulation, which together promote smooth muscle cell proliferation, fibrosis, and tumor persistence (Fartushok et al., 2025). Evidence indicates that luteolin exerts both anti-fibrotic and anti-proliferative effects in leiomyoma models. In a diethylstilbestrol (DES) + progesterone rat model, luteolin significantly reduced uterine enlargement, collagen deposition, and myometrial architectural distortion (Binmahfouz et al., 2025). These structural improvements were accompanied by restoration of antioxidant enzyme activity and suppression of lipid peroxidation (↓MDA), as well as normalization of apoptotic regulators, including an improved Bax/Bcl-2 ratio. Luteolin also attenuated pro-inflammatory mediators such as IL-6, TNF-α, and NF-κB and reduced myofibroblast activation (α-SMA), consistent with inhibition of fibrotic remodeling and restoration of redox homeostasis.

Complementary *in vitro* evidence highlights luteolin’s direct effects on leiomyoma smooth muscle cells. In studies using *Scutellaria barbata* extracts, five flavonoids, including luteolin, were isolated and evaluated for bioactivity. Among these, luteolin (alongside apigenin) demonstrated marked anti-proliferative activity in human leiomyoma smooth muscle cells while also inducing apoptosis (Kim et al., 2005). Mechanistically, luteolin reduced the expression of insulin-like growth factor-I (IGF-I) at both mRNA and protein levels. Because IGF-I is overexpressed in leiomyoma cells and drives selective tumor growth compared with normal myometrium, its downregulation represents a key pathway through which luteolin suppresses leiomyoma expansion. These findings support luteolin as a selective inhibitor of leiomyoma cell growth that acts through apoptosis activation and growth factor modulation.

### 4.6 Preeclampsia and placental dysfunction

Preeclampsia (PE) is a hypertensive disorder of pregnancy characterized by new-onset hypertension, proteinuria, and multi-organ dysfunction after 20 weeks of gestation (Martini et al., 2025). It remains a leading cause of maternal and perinatal morbidity and mortality worldwide. A central driver of PE is placental ischemia and hypoxia, which stimulate aberrant production of anti-angiogenic factors such as soluble fms-like tyrosine kinase-1 (sFlt-1), pro-inflammatory cytokines, and oxidative stress mediators (Jena et al., 2020). These signals converge on maternal endothelial dysfunction and excessive endothelin-1 (ET-1) production, driving vasoconstriction and hypertension. Given the safety limitations of pharmacologic antihypertensives during pregnancy, the exploration of nutraceuticals with anti-inflammatory and antioxidant properties has attracted increasing interest (Hup et al., 2025).

Recent studies have identified luteolin as a promising candidate capable of targeting multiple pathogenic pathways in PE. In cultured human placental cytotrophoblasts and explants from normotensive and preeclamptic pregnancies, luteolin was identified as a potent inhibitor of sFlt-1 release, reducing secretion by more than 95% compared to vehicle controls (Eddy et al., 2023). This effect occurred in a dose- and time-dependent manner and was associated with marked suppression of HIF-1α, a transcription factor upregulated in hypoxic placentae and a principal driver of sFlt-1 expression. Mechanistic experiments demonstrated that inhibition of PI3K/Akt signaling recapitulated luteolin’s effects on HIF-1α, suggesting that luteolin downregulates sFlt-1 at least in part through PI3K/Akt-dependent inhibition of HIF-1α stabilization. Follow-up studies further showed that luteolin mitigates inflammatory and oxidative stress pathways central to PE pathophysiology (Eddy et al., 2024). In human placental explants and endothelial cells stimulated with TNF-α, a cytokine elevated in PE, luteolin significantly reduced NF-κB activation, ROS, and superoxide production. Luteolin also decreased TNF-α-induced secretion of IL-6 and endothelin-1 (ET-1), the latter being a potent vasoconstrictor linked to maternal hypertension in PE. Collectively, these findings demonstrate that luteolin interrupts both inflammatory and vasoactive signaling cascades, restoring endothelial homeostasis.

### 4.7 Gynecologic malignancies

Gynecologic cancers, including ovarian, cervical, and endometrial cancers, account for around 20% of global female cancer burden (Bray et al., 2024). Despite therapeutic advances, challenges such as chemoresistance, survival of tumor stem-like cells, and metastatic spread persist. Luteolin has shown promising preclinical effects targeting several of these pathways. Cancer stem cells promote recurrence and therapeutic resistance. Luteolin directly binds KDM4C, a histone demethylase, leading to reduced stemness and tumor progression (Li et al., 2023). In CD133^+^/ALDH^+^ stem-like ovarian cancer cells, luteolin decreased sphere formation and downregulated stemness genes (SOX2, OCT4, NANOG). It also increased sensitivity to paclitaxel and carboplatin. In xenografts, luteolin (100 mg/kg IV) reduced tumor burden and prolonged survival without systemic toxicity, supporting its potential as a safe adjunct targeting tumor stemness (Li et al., 2023).

In Ca Ski cervical cancer cells, luteolin (25–100 µM) inhibited proliferation and induced intrinsic apoptosis characterized by caspase activation and mitochondrial dysfunction (Pei et al., 2024). Synergistic cytotoxic activity was observed when luteolin was combined with asiatic acid, with enhanced apoptotic effects and suppression of AKT/mTOR/NF-κB signaling (Chen et al., 2023). These data suggest luteolin as both a standalone and combination adjunct for cervical cancer therapy.

Transcriptomic analyses indicate that luteolin targets genes involved in IL-17 signaling, oxidative stress regulation, and homologous recombination repair (Zhao et al., 2023). In AN3-CA endometrial carcinoma cells, luteolin (5–15 µM) reduced migration and expression of MMP1, IL-17, and VEGF, key mediators of angiogenesis and metastasis. This supports luteolin’s potential role in modulating inflammatory and angiogenic pathways in endometrial malignancy. Collectively, the disease-specific findings summarized above are consolidated in Table 1, which outlines the key experimental models, doses, mechanisms, and outcomes across reproductive disorders.

**TABLE 1:** Summary of experimental studies investigating luteolin in female reproductive disorders.

#	Disorder	Year	Model/System	Luteolin dose and route	Duration	Key findings	References
1	PCOS (Insulin Resistant Model)	2021	Letrozole + high-fat-diet rat model (*in vivo*)	25–100 mg/kg, i.p	21 days	• Restored estrous cycle • Improved ovarian morphology • Normalized sex hormones • Improved insulin sensitivity (↓HOMA-IR) • Enhanced antioxidant status (↑SOD, CAT, GSH, GPx) • Modulated PI3K/Akt and Nrf2 pathways	Huang and Zhang (2021)
2	PCOS (Obesity-Exacerbated Model)	2025	Letrozole + high-fat-diet rat model (*in vivo*)	20–40 mg/kg, oral	28 days	• Improved estrous cycle and ovarian morphology • ↓Body weight, ↓TG, ↓TC, ↓LDL-c • Improved glucose tolerance • ↓Hepatic steatosis • Reversed 138 DEGs (UQCRC2, IRS2, NFIX, ALDH6A1) • ↑Gut microbial diversity, ↑Bacteroidota/↓Firmicutes	Dai et al. (2025)
3	PCOS-like Environmental Ovotoxicity (BPA)	2024	CHO cells (*in vitro*), and Zebrafish PCOS-like model (*in vivo*)	10–50 µM (cells) and 50 μM, i.p. (zebrafish)	24 h (cells) and 15 days (zebrafish)	• Reduced ROS and mitochondrial damage • Restored follicular maturation • Normalized ovarian morphology • ↑SOD, ↑AChE activity • ↓TNF-α, IL-1β, TOX3	Sudhakaran et al. (2024)
4	Primary Ovarian Insufficiency	2025	Cyclophosphamide-induced rat model (*in vivo*)	25–100 mg/kg, oral	28 days	• Improved follicle morphology • ↑E2, P, AMH • ↓FSH, LH • ↓Granulosa apoptosis • ↓MDA, 4-HNE, 8-OhdG	Pan et al. (2025)
5	Endometriosis	2021	Human 12Z endometriotic epithelial cells (*in vitro*), and sTHP-1–derived macrophages (*in vitro*)	1.56–100 µM	48 h	• Reduced 12Z proliferation • Induced caspase-dependent apoptosis • ↓CCL2/CCL5 (macrophage recruitment) • ↓VEGF, MMP-2/9	Woo et al. (2021)
6	Endometriosis	2019	Human endometrial/ectopic cell lines (VK2/E6E7, End1/E6E7) (*in vitro*), and mouse auto-implantation model (*in vivo*)	10–50 μM (*in vitro*) and 10 mg/kg, i.p. (*in vivo*)	24–48 h (cells) and 14 days (mice)	• ↓Lesion growth *in vivo* • Induced G0/G1 cell-cycle arrest • ↓CCNE1, CDK2, CDK4 • Inhibited PI3K/Akt and MAPK pathways • ↑Apoptosis in ectopic lesions	Park et al. (2019)
7	Endometritis	2024	*Staphylococcus* aureus-induced endometritis in mice (WT and Nrf2-KO) (*in vivo*)	10–40 mg/kg, i.p	24 h	• ↓TNF-α, IL-1β, IL-6 • ↓MPO, MDA, Fe2+ • ↑GSH, GPX4, ZO-1 • Reduced tissue injury	Gao et al. (2024)
8	Endometritis	2024	LPS-induced endometritis in Kunming mice (*in vivo*)	10–40 mg/kg, i.p. (three injections at 6-h intervals)	18 h post-LPS challenge	• ↓Uterine histopathological injury • ↓IL-1β, ↓IL-6, ↓TNF-α; ↑IL-10 • ↑SOD1, ↑CAT, ↑GPx1; ↓MDA, ↓ROS • Upregulated TLR4 expression • Inhibited NF-κB activation • Activated Nrf2 signaling	Shaukat et al. (2024)
9	Endometritis	2022	T. pyogenes biofilm assays (*in vitro*), and rat endometritis model (acetic acid + T. pyogenes inoculation) (*in vivo*)	156–312 μg/mL (*in vitro*); dose not specified (*in vivo* therapeutic study)	8 h (biofilm disruption) and treatment period per rat model protocol	• Dispersed mature T. pyogenes biofilms • ↓luxS, ↓plo, ↓rbsB, ↓lsrB expression • Reduced uterine inflammation *in vivo* • Improved endometritis severity scores	Zhang et al. (2022)
10	Uterine Leiomyomas	2025	Hormone-induced rat model (DES + progesterone) (*in vivo*)	10 mg/kg, oral	5 weeks	• ↓Uterine weight and fibrosis • Restored uterine architecture • ↑SOD, CAT; ↓MDA • Improved Bax/Bcl-2 ratio • ↓IL-6, TNF-α, NF-κB, α-SMA	Binmahfouz et al. (2025)
11	Uterine Leiomyomas	2005	Human leiomyoma smooth muscle cells and myometrial SMCs treated with Scutellaria barbata flavonoid isolates (*in vitro*)	10–50 µM	72 h	• ↓Cell proliferation • ↑Apoptosis • ↓IGF-I mRNA and protein expression • Selective inhibition of leiomyoma growth	Kim et al. (2005)
12	Preeclampsia/Placental Dysfunction	2023	Human placental cytotrophoblasts (*in vitro*), and placental explants from normotensive and preeclamptic pregnancies (*ex vivo*)	1–20 μM (*in vitro*)	24–72 h	• ↓sFlt-1 secretion • ↓HIF-1α expression • PI3K/Akt inhibition	Eddy et al. (2023)
13	Ovarian Cancer	2023	CD133+/ALDH + OCSC (Caov-3) (*in vitro*), and xenograft mice (*in vivo*)	30 µM (*in vitro*) and 100 mg/kg, IV (*in vivo*)	48 h (cells) and 21 days (mice)	• ↓Sphere formation • ↓stemness genes • ↓Tumor growth • ↑Sensitivity to paclitaxel/carboplatin	Li et al. (2023)
14	Cervical Cancer	2024	Ca Ski cells (*in vitro*)	25–100 µM	24–72 h	• ↓Cell viability • ↑Caspase-mediated apoptosis • ↓Mitochondrial membrane potential	Pei et al. (2024)
15	Cervical Cancer	2023	HeLa cells (luteolin + asiatic acid) (*in vitro*)	10–40 µM	24–48 h	• ↓Proliferation and migration • ↑Apoptosis • ↓Bcl-2, Cyclin D1, MMP-9	Chen et al. (2023)
16	Endometrial Cancer	2023	Bioinformatic model (*in silico*) and AN3-CA cells (*in vitro*)	5–15 µM	24 h	• ↓Migration • ↓MMP1, IL-17, VEGF • Identified 4-gene prognostic signature	Zhao et al. (2023)

Abbreviations: *in vitro*, cell-based experimental systems; *in vivo*, animal models; *ex vivo*, isolated human tissues studied outside the organism; *in silico*, computational analyses.

### 4.8 Cross-disease effects of luteolin on folliculogenesis and oocyte quality

Follicular development and oocyte quality depend on the integrity of the granulosa-oocyte unit, which is highly sensitive to oxidative stress, mitochondrial dysfunction, and apoptotic signaling (Wu D. et al., 2025). Beyond disease-specific outcomes, accumulating evidence indicates that luteolin modulates core mechanisms governing folliculogenesis and follicular survival. Across experimental models, luteolin consistently limits oxidative injury within granulosa cells, preserves mitochondrial function, and suppresses apoptosis, thereby reducing follicular atresia and supporting orderly follicular progression.

Mechanistically, luteolin activates antioxidant defense pathways, particularly Nrf2-regulated enzymes, while modulating PI3K/AKT signaling involved in granulosa cell survival and metabolic support of the oocyte (Ghantabpour et al., 2025). In chemotherapy- and toxin-induced ovarian injury models, luteolin preserves primordial and growing follicles, reduces granulosa cell apoptosis, and maintains circulating AMH levels, indicating stabilization of the functional ovarian reserve (Pan et al., 2025). Complementary evidence from environmental ovotoxicity models demonstrates that luteolin attenuates mitochondrial depolarization, restores follicular architecture, and improves follicular maturation, effects that are directly relevant to oocyte competence (Sudhakaran et al., 2024). Collectively, these findings support a fertility-centered role for luteolin as a modulator of follicular integrity and oocyte-supportive microenvironments, providing a mechanistic bridge between molecular signaling and reproductive potential.

## 5 Molecular pathways linking luteolin’s activity across female reproductive disorders

Luteolin exerts wide-ranging effects across female reproductive disorders through the coordinated regulation of multiple molecular pathways. It does not act through a single high-affinity receptor; instead, it exerts its protective effects through multi-target modulation of key inflammatory, oxidative, fibrotic, and proliferative pathways (Mahwish et al., 2025; Stachelska et al., 2025). It interacts weakly with receptors such as ERα/ERβ, but its principal actions arise from regulating intracellular signaling networks including NF-κB, Nrf2, PI3K/AKT, PTEN, and TGF-β/Smad. Although the disease-specific effects differ across PCOS, endometriosis, leiomyomas, POI, endometritis, and gynecologic cancers, these pathological states share overlapping signaling disturbances. Luteolin’s ability to modulate several interconnected nodes within these networks provides a mechanistic rationale for its broad therapeutic potential. This section consolidates the major molecular mechanisms through which luteolin exerts therapeutic effects, highlighting cross-talk among pathways to illustrate its systems-level regulatory profile.

### 5.1 Modulation of oxidative stress and redox homeostasis

Oxidative stress is a common driver of tissue dysfunction across reproductive disorders. It contributes to disrupted folliculogenesis in PCOS, epithelial injury in endometritis, and fibrotic remodeling in leiomyomas (Kumar and Ramanarayanan, 2025; Oyovwi et al., 2025). Luteolin restores redox homeostasis through both direct chemical antioxidant activity and activation of endogenous defense pathways. Structurally, its ortho-dihydroxy (catechol) configuration confers a strong electron-donating capacity, enabling neutralization of ROS via hydrogen-atom transfer and single-electron transfer mechanisms, while its planar conjugated system stabilizes radical intermediates (de Aguiar et al., 2025). Luteolin also chelates transition metals such as Fe^2+^ and Cu^2+^, thereby reducing Fenton-type ROS production (Ghozzi et al., 2024). Complementing these direct effects, luteolin activates the Nrf2/ARE antioxidant pathway by promoting dissociation of Nrf2 from Keap1, facilitating its nuclear translocation and induction of antioxidant genes including HO-1, NQO1, SOD, and CAT (Thiruvengadam et al., 2021). Upstream signaling through PI3K/AKT, MAPK (ERK/JNK/p38), and PKC further enhances Nrf2 stabilization and transcriptional activity. Together, these mechanisms underlie luteolin’s capacity to mitigate oxidative injury across multiple tissues: improving ovarian redox balance in PCOS (Huang and Zhang, 2021; Ghantabpour et al., 2025), preventing lipid peroxidation and ferroptosis in endometritis (Khan et al., 2024), and reducing oxidative fibrosis signaling in leiomyomas (Binmahfouz et al., 2025). By enhancing redox homeostasis, luteolin protects cellular structures, supports hormone synthesis, preserves epithelial integrity, and stabilizes mitochondrial function.

### 5.2 Suppression of pro-inflammatory signaling

Inflammation is a key pathogenic driver across reproductive disorders, promoting ovulatory dysfunction in PCOS, lesion survival in endometriosis, leukocyte infiltration in endometritis, and tumor progression in malignancies. Luteolin exerts multi-level suppression of inflammatory signaling by targeting several interconnected cascades. NF-κB is a central regulator of inflammatory gene expression (Singh et al., 2024). Luteolin suppresses this pathway by preventing phosphorylation and degradation of the inhibitory protein IκBα, thereby blocking nuclear translocation of NF-κB p65 and reducing transcription of pro-inflammatory cytokines (TNF-α, IL-1β, IL-6), iNOS, COX-2, and adhesion molecules (Khan et al., 2024). In parallel, luteolin interferes with MAPK signaling by reducing activation of ERK, JNK, and p38 kinases, key upstream amplifiers of cytokine and chemokine production (Almatroodi et al., 2024). Together, the dual inhibition of NF-κB and MAPK pathways attenuates inflammatory cascades, decreases immune cell recruitment, and disrupts the feed-forward loop sustaining chronic inflammation in reproductive tissues.

Chemokines such as CCL2 and CCL5 play essential roles in immune cell recruitment and polarization within reproductive tissues, and their dysregulation promotes chronic inflammation, macrophage infiltration, angiogenesis, and fibrotic remodeling characteristic of endometriosis and related disorders (Li et al., 2022; Guo et al., 2025). In endometriosis models, luteolin suppresses the expression of macrophage-recruiting chemokines (CCL2, CCL5) and reduces alternative (M2) macrophage polarization, thereby disrupting the macrophage-driven inflammatory and fibrotic microenvironment that supports lesion persistence (Woo et al., 2021). M2 macrophages are known to promote tissue repair, ECM deposition, angiogenesis, and lesion maintenance, and their inhibition reduces the inflammation that sustains disease progression (Guan et al., 2025; Wang et al., 2025). Such modulation alleviates the chronic inflammatory state underlying endometrial and ovarian pathologies (Zdrojkowski et al., 2023). Overall, these anti-inflammatory effects help disrupt the pathological feedback loops linking inflammation with oxidative stress, fibrosis, and hormonal imbalance.

### 5.3 Inhibition of fibrotic remodeling and regulation of apoptosis

Fibrosis is a major driver of structural distortion and functional impairment across multiple reproductive disorders, most notably uterine leiomyomas and chronic endometriosis (Vissers et al., 2024). Luteolin exerts potent anti-fibrotic activity by targeting the transforming growth factor-β (TGF-β) axis and re-establishing balanced apoptotic signaling (Wang et al., 2024). By suppressing TGF-β1 expression and preventing Smad2/3 phosphorylation, luteolin downregulates core ECM components, such as collagen I, fibronectin, and alpha-smooth muscle actin (α-SMA), thereby limiting myofibroblast activation and pathological ECM accumulation. In parallel, luteolin enhances apoptosis in aberrantly proliferative tissues through activation of caspase-3, -8, and -9, disruption of mitochondrial membrane potential, and induction of DNA fragmentation (Pei et al., 2024). Together, these anti-fibrotic and pro-apoptotic mechanisms contribute to reduced fibroid burden and ECM deposition in leiomyomas, diminished lesion density and invasiveness in endometriosis, and improved tissue homeostasis in fibrosis-associated gynecologic malignancies.

### 5.4 Modulation of the PI3K/AKT/PTEN survival and metabolic pathway

The PI3K/AKT/PTEN axis integrates metabolic control, cell survival, and growth factor responses in reproductive tissues (Matsuda et al., 2013). Dysregulation contributes to insulin resistance in PCOS, fibrotic proliferation in leiomyomas, and survival of cancer stem cells (Makker et al., 2011). Luteolin modulates PI3K/AKT signaling in a context-dependent manner, enhancing insulin-related signaling in PCOS models, while inhibiting pathological PI3K/AKT activation in fibrotic or malignant tissues (Li et al., 2016). Luteolin upregulates PTEN in leiomyoma models, counteracting PI3K-driven proliferation (Binmahfouz et al., 2025). This context-dependent modulation is beneficial, promoting cell survival in metabolic disorders (PCOS) while suppressing pathological proliferation in fibrotic conditions. Many gynecologic tumors exhibit aberrant PI3K/AKT activity (Rascio et al., 2021). Luteolin’s ability to suppress PI3K signaling while stabilizing PTEN may contribute to its antiproliferative and chemosensitizing effects in cancer models. Overall, luteolin helps restore metabolic signaling in PCOS, suppresses proliferative signaling in leiomyomas, and modulates survival pathways in cancer.

### 5.5 Modulation of estrogen and progesterone receptor signaling

Hormonal balance depends on coordinated estrogen and progesterone signaling. Disturbances, such as estrogen dominance or progesterone resistance, drive the progression of endometriosis, PCOS, and leiomyomas (Valiyevna, 2025). Molecular docking and biochemical studies indicate that luteolin interacts with estrogen receptor-α (ER-α) and estrogen receptor-β (ER-β) within their ligand-binding domains (D’Arrigo et al., 2021). This interaction produces hormone-context–dependent behavior, enabling luteolin to support estrogen-responsive gene expression in low-estrogen states while competitively limiting estrogen-driven proliferationin estrogen-dominant states. By modulating ER and PR pathways in this bidirectional manner, luteolin may correct hormonal imbalances that perpetuate reproductive pathology.

At the receptor level, luteolin’s differential binding to ER-α and ER-β allows it to function as a weak phytoestrogenic selective estrogen receptor modulator (SERM), displaying partial agonist activity in estrogen-deficient environments and antagonistic effects under estrogen-excess conditions (Maximov et al., 2013). Through this selective receptor modulation, luteolin restrains pathological estrogenic stimulation while preserving physiological endocrine signaling and endometrial differentiation, supporting its potential therapeutic value in hormone-dependent reproductive disorders.

### 5.6 Integrated cross-talk and systems-level regulation

**FIGURE 2:**
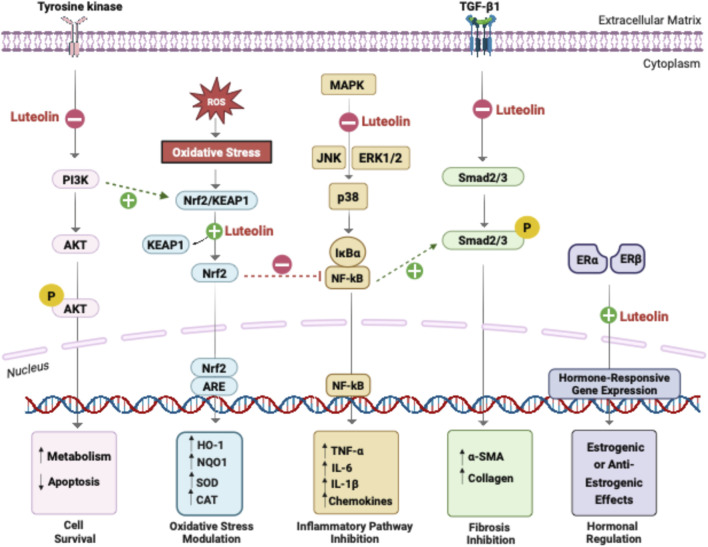
Integrated molecular pathways regulated by luteolin. Luteolin exerts multi-target regulatory effects by modulating key interconnected pathways in reproductive tissues. It suppresses PI3K/AKT activation, reduces oxidative stress through Nrf2/KEAP1 stabilization, and inhibits MAPK–NF-κB signaling, leading to decreased pro-inflammatory cytokine expression. Luteolin also attenuates TGF-β1-Smad2/3 phosphorylation to limit fibrotic responses, while interacting with ERα and ERβ to modulate hormone-responsive gene expression. Through these coordinated actions, luteolin restores redox balance, reduces inflammation and fibrosis, and regulates hormone-dependent cellular responses.

The pathways described above operate as an integrated signaling network rather than isolated linear cascades. Crosstalk between oxidative stress and inflammatory nodes is central to this network: PI3K/AKT can promote Nrf2 activation and antioxidant gene expression, while activated Nrf2 dampens NF-κB–driven transcription and limits ROS-mediated injury (Gao et al., 2022; Khassafi et al., 2024). In turn, persistent NF-κB activation upregulates TGF-β signaling, linking chronic inflammation to fibroblast activation and extracellular matrix deposition (Guo et al., 2024; Sheikh et al., 2025). TGF-β feeds back on PI3K/AKT/PTEN, shifting signaling from cytostatic responses toward pro-survival and pro-fibrotic programs in a context-dependent manner (Zhang et al., 2013). Estrogen and progesterone receptors further intersect with these nodes through rapid non-genomic activation of PI3K/AKT and MAPK cascades, thereby coupling steroid hormone status to cell survival and proliferation in reproductive tissues (Moriarty et al., 2006; Khatpe et al., 2021). By acting at these convergent signaling pathways, luteolin disrupts maladaptive redox–inflammatory–fibrotic–endocrine feedback loops and helps re-establish homeostasis in reproductive tissues, providing a systems-level explanation for its reproducible benefits across diverse gynecological disorders (Figure 2).

## 6 Luteolin products and formulation strategies

Luteolin has not yet been approved as a pharmaceutical product for any gynecological indication. However, several formulations are commercially available as dietary supplements in various international and regional markets (Table 2). Despite this broad commercial availability, none of the existing preparations are supported by clinical trials for reproductive or gynecological conditions. These products generally fall into two categories.

**TABLE 2:** Luteolin-containing supplements available in global markets.

Brand name	Formulation type	Dose	Country of manufacture	Registration classification	Key remarks
Double Wood Luteolin Supplement	Stand-alone	100 mg 120 capsules	United States	Dietary Supplement	Marketed for antioxidant and neuroprotective support
Nutricost Luteolin	Stand-alone	100 mg 120 capsules	United States	Dietary Supplement	High-purity formulation emphasizing antioxidant and immune support
aSquared Nutrition Luteolin	Stand-alone	100 mg 120 capsules	United States	Dietary Supplement	Generic capsule supporting immunity, cardiovascular health, nervous system function, and overall wellness
Neurogan Health Luteolin	Stand-alone	200 mg 120 tablets	United States	Dietary Supplement	Tablet form offers improved shelf stability; marketed for cognitive and immune wellness
Ulmubra Liposomal Luteolin	Stand-alone	800 mg 120 softgels	United States	Dietary Supplement	Liposomal formulation designed for enhanced bioavailability; marketed as a premium antioxidant supplement
MCS Formulas Luteolin Pro Liposomal	Stand-alone	150 mg 60 capsules	EU (Netherlands)	Dietary Supplement	European nutraceutical brand emphasizing liposomal delivery
Horbäach Luteolin Complex with Rutin	Combination (luteolin + rutin)	200 mg luteolin complex; 50 capsules	United States	Dietary Supplement	Combines luteolin with rutin for synergistic antioxidant action; marketed for vascular and immune health
Mirica YoungNutra (PEA + Luteolin)	Combination (luteolin + palmitoylethanolamide)	Variable ratios of luteolin and PEA	United States	Dietary Supplement	Clinically recognized formulation marketed for immune and neurological support
NeuroProtek® (Algonot LLC)	Combination (luteolin + quercetin + rutin)	100 mg luteolin 60 softgels	United States	Dietary Supplement	Clinically used neuroprotective product for cognitive function, attention, and clarity
Quicksilver scientific Liposomal Luteolin	Combination (luteolin + quercetin + DIM + vitamin C)	20 mg luteolin per 2 mL	United States	Dietary Supplement	Innovative liquid liposomal delivery enabling rapid systemic absorption. Marketed for antioxidant and histamine-modulating effects

### 6.1 Stand-alone luteolin supplements

These supplements are primarily available in capsule, tablet, or powder form, with typical dose ranges from 50 mg to 800 mg. They are marketed for general wellness benefits, including antioxidant properties, immune system support, and neuroprotective potential.

### 6.2 Combination supplements

Luteolin is often included in multiple-ingredient formulations that contain other bioactive compounds, such as flavonoids, fatty acids, or vitamins. These combination products are generally promoted for their antioxidant, anti-inflammatory, or neuroprotective properties, rather than for gynecological or endocrine-related indications.

Luteolin’s therapeutic potential is further constrained by fundamental biopharmaceutical limitations, including poor aqueous solubility, low membrane permeability, and extensive phase II metabolism, all of which restrict its oral bioavailability (Hasnat et al., 2024; Wang et al., 2024). Accordingly, formulation research has expanded from basic solubility enhancers to advanced delivery systems such as nanoemulsions, liposomes, polymeric nanoparticles, and metal–organic frameworks (Alshehri et al., 2020; Batool et al., 2020; Liu et al., 2021; Miao et al., 2021; Wu et al., 2024). However, these formulation advances have not been translated into reproductive-disorder research. In PCOS, endometriosis, and hormone-induced leiomyoma models, luteolin is almost exclusively administered in its unmodified form, typically as a simple intraperitoneal solution or oral suspension, without strategies to improve stability, absorption, or targeted delivery (Park et al., 2019; Huang and Zhang, 2021; Binmahfouz et al., 2025; Dai et al., 2025). Advanced systems, such as nano-encapsulated carriers or ligand-targeted formulations, have only been explored in gynecologic malignancies and almost entirely at the *in vitro* level, exemplified by luteolin-loaded ZIF-8 metal–organic frameworks and folate-functionalized mesoporous silica nanoparticles used in cervical cancer models (Li et al., 2022; Chen et al., 2023). This divergence underscores a clear translational gap. Although luteolin demonstrates promising biological effects across multiple reproductive pathologies, these findings rely on suboptimal pharmacokinetic conditions and non-targeted distribution. Future research incorporating optimized delivery platforms, including nanoemulsions, phytophospholipid complexes, polymeric nanoparticles, or uterus-targeted systems may substantially enhance luteolin’s therapeutic performance in reproductive medicine.

## 7 Patent landscape for luteolin in reproductive disorders

Patent analysis provides valuable insights into the developmental maturity of luteolin and the extent to which scientific interest has evolved toward commercial and therapeutic applications. Reviewing these patent filings can identify trends in innovation, highlight gaps in preclinical evidence, and evaluate the potential future direction of luteolin-based interventions in reproductive medicine. Recent years have shown a significant increase in patents related to luteolin for hormone-related and gynecological disorders. This activity is primarily concentrated in the United States, China, and South Korea, and reflects diverse therapeutic directions in gynecological and endocrine disorders (Table 3).

**TABLE 3:** International patents involving luteolin for reproductive disorders.

Country	Patent number	Indication	Formulation	Status and citation
China	CN118416049A	Obesity-related PCOS	Herbal formula containing luteolin designed to improve metabolic and endocrine dysfunction	Filed/CNIPA (Jia et al., 2024)
	CN103083310B	PCOS, dysfunctional uterine bleeding, POI, menopausal syndrome	Medicine containing luteolin as the main active ingredient for gynecological therapy	Granted/CNIPA (Wei and Ma, 2014)
South Korea	KR20210044409A	Endometriosis	Food- or supplement-based formulation combining luteolin with quercetin or delphinidin to reduce inflammation and pain related to endometriosis	Published Application/KIPO (Song Kwon and Sun-woo, 2021)
United States	US20210046042A1	Endometriosis	Pharmaceutical mixture incorporating luteolin to reduce inflammation and angiogenic signaling	Published Application/USPTO (Signorile, 2014)

Abbreviations: PCOS, polycystic ovary syndrome; CNIPA, china national intellectual property administration; USPTO, united states patent and trademark office; KIPO, korean intellectual property office; POI, primary ovarian insufficiency. Patent data compiled from Google Patents.

The patent landscape illustrates an emerging field in translational research. While international interest is increasing, most patent applications are still in the early stages, and have not progressed significantly toward investigational new drug (IND) development or clinical applications. Future patent activity will likely need to shift toward formulation engineering, targeted delivery to reproductive tissues, and the development of semi-synthetic derivatives aimed at overcoming luteolin’s pharmacokinetic limitations. Such advancements could effectively bridge the gap between promising preclinical findings and actual therapeutic options, especially for conditions like leiomyomas and diminished ovarian reserve, for which there are currently no existing patents, despite encouraging biological evidence. Collectively, the current patent landscape reflects strong commercial interest in luteolin’s anti-inflammatory and metabolic properties, yet significant opportunities remain in the areas of reproductive tissue-targeted delivery, fibrotic disorders, and fertility preservation.

## 8 Clinical studies of luteolin

Although luteolin has not yet been clinically tested in reproductive or gynecological disorders, several human studies across neuroinflammatory, neuroimmune, cognitive, and psychiatric conditions provide important insights into its biological activity, safety, and potential therapeutic relevance. In particular, formulations that combine palmitoylethanolamide (PEA) with luteolin (PEA-LUT) have undergone rigorous clinical evaluation. This body of research offers important translational evidence that aligns with the inflammatory and oxidative mechanisms associated with conditions such as PCOS, endometriosis, uterine leiomyomas, ovarian insufficiency, and endometritis.

The earliest controlled human evidence for luteolin’s neuroimmune effects comes from a randomized, double-blind clinical trial that evaluated a complex of PEA-LUT in children with autism spectrum disorder (Cordaro et al., 2020). The treatment resulted in significant improvements in behavioral scores (e.g., ABC, CARS), along with reductions in the levels of circulating IL-6 and TNF-α. Moreover, two clinical studies have examined PEA-LUT for persistent neurological and olfactory dysfunction following COVID-19. The first study was a multicenter, double-blind, randomized, placebo-controlled trial that investigated the effects of daily ultramicronized PEA-LUT (770 mg) combined with olfactory training (Di Stadio et al., 2022). This study found significantly greater improvements in olfactory threshold, discrimination, and identification compared to olfactory training alone. Complementing these results, a 3-month longitudinal study involving individuals with post-COVID-19 syndrome demonstrated that PEA-LUT, whether administered with or without prior olfactory training, significantly improved odor identification scores, reduced instances of parosmia, and alleviated mental clouding (De Luca et al., 2022). Together, these studies confirm that luteolin-containing formulations produce clinically measurable effects on neuroinflammation, neuroimmune dysregulation, mitochondrial redox imbalance, and mast cell-associated pathways. These mechanisms closely overlap with those implicated in reproductive inflammatory disorders such as PCOS, endometriosis, and uterine fibroids.

A recent double-blind, placebo-controlled crossover trial evaluated the effects of luteolin supplementation (250 mg taken twice daily for 2 weeks) on neurocognitive performance in healthy adults. The study found that luteolin led to modest yet measurable improvements in short-term and working memory (Quervain, 2024). This indicates that luteolin is bioavailable at standard oral doses and produces measurable central nervous system effects even in healthy individuals. Luteolin is currently being studied in a Phase II double-blind randomized controlled trial at the Maryland Psychiatric Research Center. In this 12-week study, individuals with schizophrenia receive either 300 mg of luteolin twice daily or a placebo. The trial aims to evaluate the effects of luteolin on psychopathology, cognitive function, oxidative stress biomarkers, and inflammatory mediators (Buchanan, 2025). This ongoing study represents a significant advance toward formal clinical development as a neuroimmune-modulating therapy.

Despite the absence of gynecology-specific clinical trials, the existing human data strongly align with the molecular and preclinical frameworks described in earlier sections, underscoring luteolin’s feasibility as a candidate for translational development in reproductive medicine. Clinical studies consistently show that formulations containing luteolin can suppress key inflammatory mediators, such as IL-6, IL-1β, TNF-α in humans. For example, open-label trials of dietary luteolin-containing supplements demonstrated reductions in serum IL-6 and TNF-α in children with autism spectrum disorders after 26 weeks of treatment (Tsilioni et al., 2015). Although large randomized controlled trials are lacking, prospective open-label human studies have shown that adaptive behavior improvements with luteolin supplementation were associated with decreased pro-inflammatory cytokines, providing translational evidence for immunomodulatory activity *in vivo* (Taliou et al., 2013). These mediators are central to the survival of endometriotic lesions, ovarian inflammation in PCOS, fibrotic signaling in leiomyomas, and epithelial injury in endometritis. Epidemiological data further suggest that higher dietary luteolin intake correlates with reduced all-cause and cardiovascular mortality, indicating broader metabolic and inflammatory benefits of luteolin intake in human populations (Yao and Zhou, 2024). Together, these findings provide a strong biological rationale for advancing luteolin into human clinical trials targeting female reproductive disorders.

## 9 Tolerability, safety and regulatory considerations

Preclinical investigations consistently demonstrate that luteolin is well tolerated across multiple experimental systems. In rodent studies, oral dosing has shown a wide margin of tolerance, with no adverse alterations in hematological or biochemical parameters even at repeated administrations, and only transient gastrointestinal effects reported at higher exposures (Abdrabou et al., 2024). Comparable findings were observed in mice, where systemic administration did not produce detectable hepatic or renal abnormalities, and histological examination confirmed preserved tissue architecture following multi-week treatment protocols (Mugale et al., 2024). Complementary *in vitro* assessments further indicate that luteolin exhibits minimal cytotoxicity toward non-malignant cells at concentrations typically required for anti-inflammatory or antioxidant actions, supporting a favorable biological response profile (Tuli et al., 2022). Short-term human data also support luteolin’s tolerability. Clinical studies using ultramicronized PEA-LUT have administered 100–600 mg/day luteolin equivalents for 2–12 weeks without any serious adverse events (Cordaro et al., 2020; De Luca et al., 2022; Di Stadio et al., 2022). Trials consistently report only mild, transient gastrointestinal discomfort or headaches, with no hepatotoxic, nephrotoxic, hematologic, or systemic toxicity. A placebo-controlled trial administering 250 mg luteolin twice daily in healthy adults likewise showed excellent tolerability with no clinically meaningful adverse events (Quervain, 2024).

Several pharmacokinetic considerations warrant attention. Luteolin undergoes extensive UGT- and SULT-mediated phase II metabolism, raising the potential for drug-drug interactions with medications using the same metabolic pathways (Quintieri et al., 2008; Wang et al., 2024). *In vitro* assays also document inhibitory effects on CYP1A2, CYP2C9, and CYP3A4, suggesting that high-dose or chronic exposure may alter the pharmacokinetics of drugs with narrow therapeutic windows (Kaci et al., 2023). Although the clinical significance of these interactions remains undetermined, these findings highlight the need for formal pharmacokinetic studies.

From a regulatory standpoint, luteolin-containing products are globally classified as dietary or nutraceutical supplements rather than therapeutic agents. In the United States, they fall under the Dietary Supplement Health and Education Act (DSHEA) of 1994, and do not require FDA pre-market approval. In Saudi Arabia, the Saudi Food and Drug Authority (SFDA) regulates luteolin-based products as food supplements in accordance with the Products Classification Guidance (Version 7, 2024). The European Food Safety Authority (EFSA) lists luteolin as a conventional botanical ingredient with no approved health claims. Similarly, Health Canada categorizes it as a natural health product and the Australian Therapeutic Goods Administration (TGA) includes it among permissible ingredients for complementary medicines. Overall, luteolin is well tolerated in animals and humans, with a low incidence of adverse events at clinically relevant doses. Nonetheless, rigorous GLP-compliant toxicology, drug-interaction studies, and reproductive health–focused clinical trials are needed to fully define long-term safety and support its development for gynecologic indications.

## 10 Conclusions and future directions

Female reproductive disorders remain a major global health challenge, with limited innovative therapeutic options, underscoring the urgent need for multi-target agents capable of addressing the intertwined endocrine, metabolic, inflammatory, and fibrotic pathways that drive disease progression. To our knowledge, this review provides the most integrated synthesis to date of luteolin’s chemical foundations, mechanistic actions, disease-specific effects, formulation advances, regulatory status, and translational implications.

Luteolin has been shown to play a significant role in various reproductive disorders, such as PCOS, endometriosis, uterine leiomyomas, POI, endometritis, and gynecologic malignancies. It consistently modulates common pathological processes including oxidative stress, inflammation, fibrosis, metabolic dysfunction, abnormal apoptosis, and hormonal imbalance (Huang and Zhang, 2021; Woo et al., 2021; Gao et al., 2024; Binmahfouz et al., 2025; Pan et al., 2025). From a mechanistic standpoint, luteolin affects multiple interconnected pathways including the Nrf2/ARE antioxidant pathway, NF-κB and MAPK inflammatory cascades, PI3K/AKT/PTEN metabolic-survival signaling, TGF-β/Smad fibrotic pathways, and ER/PR hormonal regulation (Thiruvengadam et al., 2021; Khan et al., 2024). These comprehensive actions highlight luteolin’s potential as a systems-level regulator that can restore balance among redox, immune, and endocrine functions. Recent advances in formulations such as lipid-based systems, polymeric micelles, β-cyclodextrin-metal-organic frameworks (MOF), and luteolin prodrugs have shown considerable promise in enhancing solubility, oral bioavailability, metabolic stability, and tissue exposure (Liu et al., 2021; Miao et al., 2021; Wu et al., 2024; Yang et al., 2024). Growing patent activity further reflects translational interest, although major gaps remain in targeted delivery and leiomyoma- or fertility-specific applications. Early human studies from neuroimmune and post-COVID populations demonstrate luteolin’s anti-inflammatory and antioxidant activity (Cordaro et al., 2020; De Luca et al., 2022; Di Stadio et al., 2022), suggesting potential relevance to reproductive disorders characterized by similar inflammatory profiles.

**FIGURE 3:**
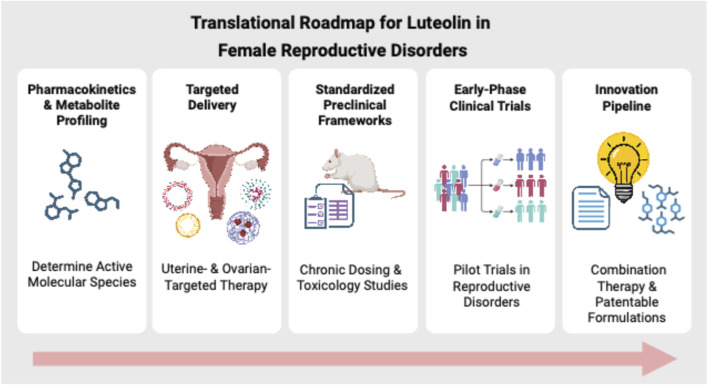
Translational roadmap for luteolin in female reproductive disorders. A schematic summary of the key steps necessary to advance luteolin toward clinical application. It includes pharmacokinetic and metabolite profiling, targeted delivery strategies for the uterus and ovaries, standardized preclinical models, and early-phase clinical trials for conditions such as PCOS, endometriosis, and leiomyoma. Additionally, it highlights innovations through the development of prodrugs, combination therapies, and patentable formulations.

To advance luteolin toward clinical application, future research should follow a structured translational roadmap. First, comprehensive pharmacokinetic and metabolite-profiling studies are needed to identify whether therapeutic activity is driven primarily by the aglycone form or its glucuronidated and sulfated metabolites. Parallel efforts should prioritize uterus- and ovary-targeted delivery platforms, including nanocarriers, lipid systems, and prodrug strategies, to enhance tissue specificity and reduce interindividual variability. Standardizing preclinical models, dosing strategies, biomarkers, and follow-up durations will be essential for improving reproducibility and supporting meta-analytic interpretation. Building on these foundations, early-phase clinical trials in PCOS, endometriosis, and leiomyomas should prioritize subgroups with high oxidative, inflammatory, metabolic, or fibrotic burden. Finally, expanding the innovation pipeline through prodrug development, synergistic combination therapies, and patentable formulation strategies will help overcome current pharmacokinetic challenges and accelerate translation. This roadmap (Figure 3) highlights the essential steps needed to advance luteolin from promising preclinical evidence toward human therapeutic development.

In conclusion, luteolin is a biologically versatile flavone with a complex mechanism of action, showing significant potential to address unmet therapeutic needs in female reproductive medicine. Its consistent efficacy across preclinical models, together with emerging translational evidence, provides a strong rationale for further investigation. Future progress will depend on integrating advances in pharmacology, formulation science, reproductive biology, and clinical research. With sustained interdisciplinary collaboration, luteolin has the potential to evolve from an underrecognized nutraceutical into a rigorously validated therapeutic platform with meaningful impact on women’s reproductive health and quality of life.

## Glossary

4-HNE: 4-Hydroxynonenal
8-OHdG: 8-Hydroxy-2′-deoxyguanosine
ABC: Aberrant Behavior Checklist
AChE: Acetylcholinesterase
AKT: Protein Kinase B
ALDH: Aldehyde Dehydrogenase
AMH: Anti-Müllerian Hormone
AN3-CA: Human Endometrial Carcinoma Cell Line
ARE: Antioxidant Response Element
ASD: Autism Spectrum Disorder
ATP: Adenosine Triphosphate
Bax: BCL2-Associated X Protein
BCS: Biopharmaceutics Classification System
Bcl-2: B-Cell Lymphoma 2
BPA: Bisphenol A
CARS: Childhood Autism Rating Scale
CAT: Catalase
CCL2: C-C Motif Chemokine Ligand 2
CCL5: C-C Motif Chemokine Ligand 5
**CD133** ^**+**^: Prominin-1 (Cancer Stem Cell Marker)
CHO: Chinese Hamster Ovary
COX-2: Cyclooxygenase-2
COVID-19: Coronavirus Disease 2019
CSC: Cancer Stem Cell
DEGs: Differentially Expressed Genes
DES: Diethylstilbestrol
DIM: 3,3′-Diindolylmethane
DNA: Deoxyribonucleic Acid
ECM: Extracellular Matrix
EFSA: European Food Safety Authority
ER: Estrogen Receptor
ER-α: Estrogen Receptor-Alpha
ER-β: Estrogen Receptor-Beta
ERK: Extracellular Signal-Regulated Kinase
ET-1: Endothelin-1
**Fe** ^**2+**^: Ferrous Iron
FSH: Follicle-Stimulating Hormone
GPX1: Glutathione Peroxidase 1
GPX4: Glutathione Peroxidase 4
GSH: Reduced Glutathione
HIF-1α: Hypoxia-Inducible Factor 1-Alpha
HOMA-IR: Homeostatic Model Assessment for Insulin Resistance
HO-1: Heme Oxygenase-1
HPLC: High-Performance Liquid Chromatography
IGF-I: Insulin-Like Growth Factor I
IL-1β: Interleukin-1 Beta
IL-6: Interleukin-6
IL-17: Interleukin-17
iNOS: Inducible Nitric Oxide Synthase
i.p.: Intraperitoneal
IV: Intravenous
**J**NK: c-Jun N-terminal Kinase
KDM4C: Lysine Demethylase 4C
Keap1: Kelch-like ECH-Associated Protein 1
KO: Knockout
LDL-c: Low-Density Lipoprotein Cholesterol
LH: Luteinizing Hormone
LPS: Lipopolysaccharide
MAPK: Mitogen-Activated Protein Kinase
MDA: Malondialdehyde
MMP: Matrix Metalloproteinase
MOF: Metal–Organic Framework
MPO: Myeloperoxidase
mTOR: Mammalian Target of Rapamycin
NHANES: National Health and Nutrition Examination Survey
NF-κB: Nuclear Factor Kappa-Light-Chain-Enhancer of Activated B Cells
NQO1: NAD(P)H Quinone Dehydrogenase 1
Nrf2: Nuclear Factor Erythroid 2–Related Factor 2
OCSC: Ovarian Cancer Stem Cell
PCOS: Polycystic Ovary Syndrome
PEA: Palmitoylethanolamide
PEA-LUT: Palmitoylethanolamide–Luteolin
POF: Premature Ovarian Failure
PPARγ: Peroxisome Proliferator-Activated Receptor Gamma
PPP2CA: Protein Phosphatase 2 Catalytic Subunit Alpha
PR: Progesterone Receptor
PR-B: Progesterone Receptor-B
PTEN: Phosphatase and Tensin Homolog
ROS: Reactive Oxygen Species
SOD: Superoxide Dismutase
SNEDDS: Self-Nanoemulsifying Drug Delivery System
S-SNEDDS: Supersaturable Self-Nanoemulsifying Drug Delivery System
SERMs: Selective Estrogen Receptor Modulators
sFlt-1: Soluble Fms-like Tyrosine Kinase-1
SULTs: Sulfotransferases
TGA: Therapeutic Goods Administration
TG: Triglycerides
TC: Total Cholesterol
TGF-β: Transforming Growth Factor Beta
TLR4: Toll-Like Receptor 4
TNF-α: Tumor Necrosis Factor Alpha
TOX3: TOX High Mobility Group Box Family Member 3
UGTs: UDP-Glucuronosyltransferases
USA: United States of America
VEGF: Vascular Endothelial Growth Factor
YAP: Yes-Associated Protein
ZO-1: Zonula Occludens-1
